# Molecular targets for rapid identification of *Brucella *spp

**DOI:** 10.1186/1471-2180-6-13

**Published:** 2006-02-22

**Authors:** Vladyslava G Ratushna, David M Sturgill, Sheela Ramamoorthy, Sherry A Reichow, Yongqun He, Raju Lathigra, Nammalwar Sriranganathan, Shirley M Halling, Stephen M Boyle, Cynthia J Gibas

**Affiliations:** 1 Department of Computer Science, College of Information Technology, University of North Carolina at Charlotte, Charlotte, NC, 28223, USA; 2 Department of Biology, College of Science, Virginia Polytechnic Institute & State University, Blacksburg, VA, 24061, USA; 3 Virginia-Maryland Regional College of Veterinary Medicine, Virginia Polytechnic Institute & State University, Blacksburg, VA, 24061, USA; 4 Unit for Laboratory Animal Medicine, School of Medicine, University of Michigan, Ann Arbor, MI 48105, USA; 5 Walter Reed Army Institute of Research, Department of Bacterial Diseases, Division of Communicable Diseases and Immunology, 503 Robert Grant Avenue, Silver Spring, MD 20910, USA; 6 Bacterial Diseases of Livestock Research Unit, United States Department of Agriculture, Agricultural Research Service, National Animal Disease Center, 2300 Dayton Rd, Ames, IA, 50010, USA

## Abstract

**Background:**

*Brucella *is an intracellular pathogen capable of infecting animals and humans. There are six recognized species of *Brucella *that differ in their host preference. The genomes of the three *Brucella *species have been recently sequenced. Comparison of the three revealed over 98% sequence similarity at the protein level and enabled computational identification of common and differentiating genes. We validated these computational predictions and examined the expression patterns of the putative unique and differentiating genes, using genomic and reverse transcription PCR. We then screened a set of differentiating genes against classical *Brucella *biovars and showed the applicability of these regions in the design of diagnostic tests.

**Results:**

We have identified and tested set of molecular targets that are associated in unique patterns with each of the sequenced *Brucella *spp. A comprehensive comparison was made among the published genome sequences of *B. abortus, B. melitensis *and *B. suis*. The comparison confirmed published differences between the three *Brucella *genomes, and identified subsets of features that were predicted to be of interest in a functional comparison of *B. melitensis *and *B. suis *to *B. abortus*. Differentiating sequence regions from *B. abortus, B. melitensis *and *B. suis *were used to develop PCR primers to test for the existence and *in vitro *transcription of these genes in these species. Only *B. suis *is found to have a significant number of unique genes, but combinations of genes and regions that exist in only two out of three genomes and are therefore useful for diagnostics were identified and confirmed.

**Conclusion:**

Although not all of the differentiating genes identified were transcribed under steady state conditions, a group of genes sufficient to discriminate unambiguously between *B. suis*, *B. melitensis*, and *B. abortus *was identified. We present an overview of these genomic differences and the use of these features to discriminate among a number of *Brucella *biovars.

## Background

*Brucella *is a facultative intracellular pathogen that causes abortion in cattle, goats and sheep and a febrile illness ("undulant fever") in humans. Animal brucellosis is a serious problem worldwide and is endemic globally. In areas where it is endemic, human brucellosis is quite common but often not diagnosed. There are six recognized *Brucella *species that differ in their preference for certain hosts. *B. abortus *preferentially infects cattle, *B. melitensis *infects sheep and goats, and *B. suis *infects pigs. All three of these species, as well as *B. canis*, can infect humans, and *B. melitensis *is associated with the most serious human infections [1,2]. The brucellae are grouped with the α-proteobacteria and are related to other cell-associated parasites of plants and animals. The classical *Brucella *taxonomy is based on six species (*B. melitensis*, *B. abortus*, *B. suis*, *B. neotomae*, *B. ovis *and *B. canis*) characterized by their host preferences. Later observations of high homology from DNA-DNA hybridization studies led many to adopt a monospecific system [3,4]. The Subcommittee on the Taxonomy of Brucella also accepted this classification in 1986, along with the caveat that the classical species names should be used "to avoid confusion." Most researchers still prefer to use the species system, which recently has been given more credence by detailed biochemical and genetic studies [5].

Macrophages are among the first targets of *Brucella *invasion, and the bacteria can survive within this naturally hostile intracellular environment [6]. Macrophages are important in transporting *Brucella *to tissues throughout the host, where they can survive in a variety of cell types [7]. Several studies have suggested that *Brucella *delays phagolysosomal fusion as a survival mechanism in macrophages, while in non-professional phagocytes *Brucella *appears to modulate the interior of the phagosome and evades intracellular degradation by avoiding the endocytic/phagocytic cascade [8]. It is not known definitively where *Brucella *replicates within the vertebrate cell. Observations have suggested that *Brucella *replicates within the rough endoplasmic reticulum (ER) in several cell types, including trophoblasts [9] and Vero cells [10]. It has been shown that lipid raft-associated molecules, such as glycophosphatidylinositol anchored molecules, play a role in determining the intracellular fate of *Brucella *[11]. Studies identifying ER markers on *Brucella*-containing compartments have also supported the theory of the ER as the site of replication [8]. The detailed mechanism of *Brucella *intracellular survival is not well understood and is assumed related to patterns of gene expression in both the pathogen and host. For example, most *Brucella spp*. are smooth due to expression of O-side chain genes, replicate inside macrophages and are virulent. In contrast, *B. canis *is rough, yet still capable of macrophage survival and is also virulent [12,13]. In the interest of developing a DNA microarray optimized for comparative study of the brucellae, we have carried out a three-way genome comparison of *B. suis *[14], *B. melitensis *[15], and draft *B. abortus *[16] sequences, at both the nucleotide and predicted coding sequence (CDS) levels. *B. melitensis*, *B. suis*, and *B. abortus *each have approximately 3 Mb of genomic DNA, divided into a large chromosome of approximately 2 Mb and a small chromosome of approximately 1 Mb. We found that over 3100 genes identified in *B. suis *or *B. melitensis *appear to have a homolog in both of these genomes and also in *B. abortus*. Fewer than 100 genes were identified as present in only one or two of the three genomes, with an additional group of close to 100 genes having significant deletions in one or two of the genomes relative to the others. Annotated or predicted genomic sequence features that appear to distinguish the three species were probed using PCR and RT-PCR, to verify their uniqueness and to test for transcription under *in vitro *growth conditions. PCR primers were then used to assay and classify several variant strains. Differentiating genes will provide targets for rapid discrimination among *Brucella *species, as probes on a diagnostic chip, and will also be useful for elucidation of differences in host preference and mechanism of virulence among these closely related species.

## Results and discussion

### Three-way genome comparison

The genomes of the three sequenced Brucella biovars (*B. abortus *9-941, *B. melitensis *16 M and *B. suis *1330) were compared globally to identify the extent of similarity between these closely related bacterial species.

**Figure 1:**
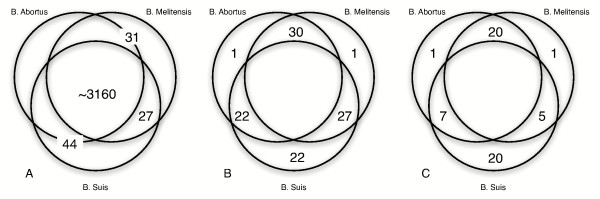
**Distribution of differentiating genes in three *Brucella *sequences**. The figure contains Venn diagrams, showing the distribution of differentiating genes in the three *Brucella *genomes: A) predicted from whole-genome sequence comparison of *B. melitensis *16 M and *B. suis *1330 with additional publicly available sequence from *B. abortus *9-941 B) confirmed by genomic PCR analysis and C) shown to be transcribed by RT-PCR analysis

Our results for comparison of *B. suis *to *B. melitensis *(Figure 1A) were generally in agreement with those of Paulsen *et al*., 2002 [14]. We further tried to pinpoint which of the potentially unique genes could also distinguish *B. melitensis *and *B. suis *from *B. abortus*, and would therefore be of interest as differentiating probes on an expression array, by including draft genome sequence from *B. abortus *in the comparison. Subsequent results of comparisons to the finished *B. abortus *sequence by Halling et al. [16], are in reasonable agreement with our results, though some minor differences in feature identification arose from our use of a draft annotation. Our computational and experimental analysis identified and confirmed a set of 22 ORFs to be present in *B. suis *1330, but not in *B. melitensis *16 M or *B. abortus *9-941, and another 22 ORFs found in both *B. suis *1330 and *B. abortus 9-941*, but not in *B. melitensis *16 M. These differentiating ORFs extend a known set of 217 ORFs, which have been shown experimentally to differ in expression between the *Brucella *species in a *B. melitensis *16 M based microarray experiment [17]. Our three-way genomic comparison together with the genomic comparison performed by Halling et al. [16], demonstrates that *B. suis *1330 and *B. abortus *9-941 both contain the ORFs identical to BMEI1747, BMEII1071, BMEI 1746, and BMEI1896, BMEI1919-21, BMEI1923, BMEII0826, BMEII0850, and BMEI1662, which were shown to be completely or partially missing from either *B. suis *S100 or *B. abortus *S2308 or both respectively [17].

Supporting previous findings that the three genomes are highly similar, the majority (>90%) of annotated genes were found to share 98–100% sequence identity at the nucleotide level with their apparent homologues in each of the other genomes. The majority of differentiating genes identified are in large (~20 kb) regions, which partly account for differences in chromosome size. Most of these genes have functional assignments in existing annotation. Table 1 provides a detailed description of the differentiating genes identified in this study, organized according to their order in the *B. suis *genome, or in the *B. melitensis *genome in those cases where there was no *B. suis *sequence match. Genes shown as present in *B. suis *and *B. melitensis *but absent in *B. abortus *mainly correspond to a large deletion in the genome sequence of *B. abortus *relative to the other genomes, which was previously identified by Vizcaino and colleagues [18], and identifiable in [GenBank:AF076290]. The identities of these genes were confirmed by comparison of that record to the draft *B. abortus *sequence.

### Functional significance of genomic differences

We identified several multi-gene regions that contain the majority of differentiating genes (Table 2). These six regions alone are sufficient to discriminate between the three *Brucella *species. In a pairwise comparison, thirty-three regions were described as unique to either *B. suis *or *B. melitensis *[14]. In a three-way comparison that included the draft *B. abortus *sequence, we find that many of these differentiating features can no longer be considered unique for the purpose of discriminating among the three species. Fewer single-species specific genes remain, twenty-two unique genes in *B. suis *and one in *B. melitensis*, which demonstrates the homogeneity of the genus. A complete list of differentiating coding regions is given in Table 1, and their possible significance is described below.

#### Metabolism

Several CDSs homologous to components of an amino acid ABC transport system were found in *B. abortus *and *B. suis *but were absent in *B. melitensis*. This may indicate that *B. abortus *and *B. suis *have the ability to utilize a nutrient that *B. melitensis *does not. Different patterns of amino acid utilization are used to distinguish among the brucellae [19], and variations in amino acid transporter content are consistent with the observation that each species has a distinct pattern of nutrient utilization. Most of these genes are present on the differentiating region SA2 (Table 2), suggesting that the acquisition or loss of this region could have been related to a change in environment or nutrient availability for the ancestral species. Two ABC transporter permeases (BR0952/BR0953) unique to *B. suis *were also identified. Transcription of these genes in *B. suis *was detected by RT-PCR (Table 3).

#### Virulence

A detailed analysis of a 50 kb region (BRA1072-1116/BMEII0183-227) was performed to complement our general comparison of gene content. This 50 kb region resides on Chromosome II of each *Brucella *species and may represent a composite transposon [14]. It is flanked with insertion sequences that suggest a foreign origin, although its G+C content (56.8%) is close to the *Brucella *average. Although this island does not contain obvious virulence genes, it includes a large number of peptide ABC transporter genes. In some pathogens, autotransporter proteins have been implicated as virulence determinants [20]; whether this is the case for the brucellae has not been reported as yet.

Comparison with *B. suis *shows that this region is also present in *B. melitensis *and *B. abortus *(Bricker, Acc. No. AF454951) but contains deletions in the dipeptide ABC transporter permease protein gene, the 3-hydroxyacyl-CoA dehydrogenase family protein gene, and a transcriptional regulator. Each of these small deletions is in-frame, but result in missing amino acids and potentially in altered function, perhaps explaining significant metabolic differences between the three species.

A 25 kb region present only in *B. suis *and *B. melitensis *was revealed by three-way comparison to be a differentiating feature. This region, absent only in *B. abortus *(region MS2, Table 2), contains five glycosyl transferases (BMEII0835/0837/0840/0845-0847; BRA0420-0422/0427/0430/0432) and a succinoglycan biosynthesis transport protein (BMEII0838/BRA0429). However, no transcription of succinoglycan biosynthesis transport protein was detected by RT-PCR for either species. In *B. melitensis*, transcription of four out of five glycosyl transferases was detected by RT-PCR, while in *B. suis *transcription of only one of these genes was observed. These genes may be important in O-side chain biosynthesis – one of the known virulence determinants of *Brucella *[21]. This region also contains several uncharacterized genes that may be novel virulence factors, including a putative outer membrane protein and several conserved hypothetical proteins. This region was shown to be present in *B. melitensis*, *B. suis*, *B. ovis*, *B. canis*, and *B. neotomae*, but not in *B. abortus *[18]. Vizcaino *et al*. conjecture that this region is absent due to a deletion event before the differentiation of this species and its biovars, since none of the *B. abortus *biovars possess this region. The deletion of this island may have impacted the host range of *B. abortus *and pushed its divergence from the *Brucella *ancestor.

A three-way comparison also reveals species-specific differences in two gene clusters of urease subunits present on Chromosome II of *B. suis*, *B. abortus*, and *B. melitensis *(*ure*A-G-1 BR0267-BR0273 and *ure*A-G-2 BR1356-BR1362 in *B. suis*). Some subunits of these clusters are conserved among other bacterial species, and ureases have been shown to be important to virulence in several animal models of bacterial infection [22]. *B. melitensis *has a 1 bp insertion in *ure*A-1 (BR0268), representing a potential frame shift. A 6 bp insertion in the *ure*D-2 (BR1362) gene of *B. abortus *was identified, within overlapping segments of a highly repetitive region of the gene. In the *ure*E-2 gene (BR1359) of *B. abortus *two separate single base deletions are present, possibly shifting the frame of translation. Finally, the last 22 bp of *ure*E-1 (BR0271) were shown to be 100% identical in *B. abortus *and *B. melitensis *but significantly diverged in *B. suis*, including a 2 bp deletion. This variation within these urease gene clusters could prove to be significant to virulence differences.

#### Secretion systems

Our analysis revealed a cluster of transfer genes (*tra/trb*) unique to *B. suis *and potentially significant to secretion (region S2, Table 2). Transcription of all but one gene in this island was observed by RT-PCR. Several genes in this region (*trbL*, *trbJ*, *traC*, *traJ*, traI, and *repA*) are homologous to genes involved in mating pair formation described for *Escherichia coli *plasmid RP4 [23], to receptor complex formation in bacteriophage-host gene transfer systems [24], and to genes of type IV secretion systems of other species of bacteria. *Agrobacterium *contains both a *vir*B type IV secretion system and a *tra/trb *bacterial conjugation system. These systems are homologous and share common ancestral origins, but they are functionally independent and physically separate [25,26]. *Brucella *spp. lack a conjugation system, which suggests that the genes in this region play a role in type IV secretion, or are part of an uncharacterized macromolecule or gene transfer system. The organization of this unique S2 region suggests a pattern of co-transcription. The short intergenic regions between the CDSs may indicate that these genes are organized as operons and are co-transcribed. In the case of the BRA0372-BRA0373 operon, the start codon of BRA0373 lies within BRA0372 that may indicate a -1 or -2 frame shift mechanism for transcription of BRA0373. Examples of this type of gene/operon organization have primarily been identified in viruses [22,27]. It has also been identified in prokaryotes [28], although in some cases it can be an artifact of annotation error [29]. Additional study is needed to confirm the annotation in this case. Type III secretion systems are assembled from components of flagellar machinery [30]. Although *Brucella *does not normally produce flagella, our analysis reveals a flagellar gene (FlgJ – BMEI1692) present in differentiating region MA1. This gene is on Chromosome I, instead of within one of three flagellar gene clusters on Chromosome II. It is also more than twice (~640 aa) the normal size (~313 aa) for this protein. In *B. melitensis*, all the structural genes for flagellum formation are present but genes for the chemotactic receptors or transducers are absent [31]. Based on the presence of several flagellar genes and a homologue of the LcrD virulence superfamily in *B. abortus*, it has been suggested that *Brucella *has the potential for motility and type III secretion [32]. However, a recent study did not detect transcription by RT-PCR in *B. melitensis *grown in Albimi broth of four flagellar genes (*flhB*, *flhP*, *fliR*, *fliF*) present in *B. suis*, *B. abortus*, and *B. ovis *[33]. Our RT-PCR results revealed no transcription of the flagellar differential gene *flg*J in *Brucella *grown in trypticase soy broth. Transcription was detected in ten genes within the same region MA1 that are defined as hypothetical proteins [31]. Recent studies suggest that a flagellar gene promoter (*fliF*) is induced when *B. suis *is replicating in macrophages; additional studies on flagellar gene expression have been performed [34,35]. It appears that at least *B. melitensis *can produce flagella transiently *in-vitro *in pure culture [35] and fla genes are necessary for chronic infection in mice [34]. Thus it is likely that flagellar gene expression occurs when *Brucella *is replicating in an intracellular environment such as macrophages.

#### Site-specific recombinases

An apparent recombinase homologue (BMEI1661) was identified as the sole unique gene for *B. melitensis*, and our RT-PCR results indicated that it is transcribed. There are two resolvase family genes (BME1661/BMEI0902) in the *B. melitensis *annotation for Chromosome I located in opposite orientations. These two genes share homology over a 180 bp consensus sequence. However, one putative recombinase (BMEI1661) is much larger than the other (747 bp vs. 231 bp). They may be considered paralogous, but BME1661 contains more than 500 bp not present in any other species. In the *B. suis *annotation, there are two almost identical recombinases of equal size (617 bp) present, in opposite orientations. These only have small matches to BME1661/BMEI0902 (~40 bp). However, both *B. abortus *and *B. suis *contain 2 copies of ~180 bp homologous to BME1661/BMEI0902, mostly within intergenic sequence. Overall, a 180 bp consensus is present in two copies in all three species, but ~500 bp of the BMEI1661 gene in *B. melitensis *is unique to this species. Site-specific recombination has been shown to be involved with acquisition of drug resistance genes and alteration of gene expression [36], suggesting that this unique gene may play a role in virulence.

#### Evolutionary implications

Our analysis reinforces the view that the brucellae are highly similar – much more identical to each other than are other groups of closely related bacteria. It has been suggested that the low rate of genetic exchange between *Brucella spp*. and other species is due to their niches within cells as intracellular parasites [37]. However, several multi-gene differentiating islands identified in our comparison (Table 2) contain atypical G+C contents that is consistent with gene acquisition via horizontal transfer. Island MA1 exhibits a G+C content of 52% and contains a putative phage integrase family transposase at the end of the gene cluster in both *B. abortus *and *B. melitensis*. *Escherichia coli *has a G+C content of 51.4%, and has been demonstrated to transfer a broad host range plasmid to *Brucella *under laboratory conditions [38]. Other islands have base compositions close to the average *Brucella *G+C content. Island SM2 exhibits a G+C content of 58% in both *B. melitensis *and *B. suis*. The presence of phage genes suggests that lysogenic conversion may have occurred [39]. The island S2 that is unique to B. suis and containing 5 tra/trb genes has a G+C content of 55.6% and is flanked by a phage integrase homologue. Two phage gene homologues (a HK97 family phage major capsid protein and putative phage head-tail adaptor) are present within island SA1 and two phage gene homologues (a HK97 family portal protein and a phage terminase subunit) flank the island. Island SA2 contains a phage minor tail protein L homologue. This evidence is consistent with phage-mediated transduction and suggests that phages may have helped the brucellae adapt to their intracellular niches.

### RT-PCR analysis of differentiating regions

Reverse transcription (RT-PCR) experiments were performed for all of the predicted coding sequences from the differentiating regions of *B. suis, B. melitensis *and *B. abortus *to determine whether they are transcribed in the species-specific pattern expected. When no amplicon was observed by RT-PCR, regular PCR reactions were performed to confirm the presence of differentiating sequences in genomic DNA. A total of 105 primer pairs were designed, for RT-PCR reactions targeting a total of 102 genes. Three of these were partial differentials – homologs that appeared to have significant insertions in one species relative to another in which a unique primer or probe could be placed to distinguish among species – and more than one primer pair was used in some of these cases.

Figure 1 is a Venn diagram of gene content in the three species. The separate sections of the figure show (A) the number of differentiating genes identified by sequence comparison and (B) confirmed in the genome sequence by PCR, and (C) transcribed *in vitro*, as detected by RT-PCR. The smaller number of differentiating features in Figure 1C relative to Figure 1B signifies only that some genes which we identified as differentiating features were not transcribed under the conditions of this experiment – *in vitro *and in late log phase.

Table 2 summarizes transcripts detected for genes in each differentiating sequence island, and Table 3 shows details of predicted vs. observed RT-PCR results for each differential gene when laboratory strains of *B. suis*, *B. melitensis*, and *B. abortus *were probed. The RT-PCR results agreed with the results of the comparative analysis. PCR amplification of the genomic DNA confirmed the presence of the DNA segment in all cases where transcription was not detected. No unexplained transcription was detected in any case where we had predicted that the gene probed would be absent. Further work will be required to determine if differentiating genes are transcribed in the intracellular environment, e.g. the macrophage, and what effect their transcription has on the ability of the *Brucella *to replicate inside macrophages.

### Differential targets identify variant strains

PCR and RT-PCR assays for predicted differential genes in the sequenced *Brucella *biovars showed that these differentials do occur in the predicted patterns. They can be used to discriminate genomic DNA from isolates of the three sequenced strains, although not all of the differentials are expressed in the late log phase. To test whether the differential sequences would be useful distinguishing the sequenced strains among a larger field of *Brucella *biovars we assayed 18 biovars using 24 of the 102 primer pairs.

**Figure 2:**
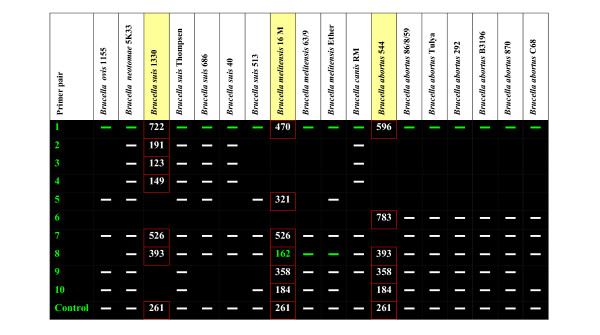
**Graphical panel of PCR results in 18 *Brucella *biovars**. The panel represents the patterns observed when PCR screening the differential regions across the 18 classical *Brucella *biovars and provides the values for the expected amplicon sizes. * The PCR was performed on extracted genomic DNA, rather than whole cells; green color shows size variability; red boundary indicates expected PCR amplification.

We found that ten of the PCR primer pairs tested could provide information about strain identification when other biovars were considered. The PCR results are summarized in a graphical panel on Figure 2, and the primer sequences and amplicon sizes are provided in Table 4. The presence of an amplicon from primer pair 6 is uniquely characteristic of the *B. abortus *strains [16]. This primer pair was screened against the entire GenBank Database and turned to be highly *B. abortus *specific. The presence of amplicons from pairs 2, 3 and 4 is characteristic of all *B. suis *strains except 513. These sequences are also present in *B. canis*; additional identifying information is provided by the variable region amplified by probe pair one, as described below. Primer pair 5 was originally selected to identify *B. melitensis*, but was found to occur in some *B. suis *strains. However, primer pair 8 was able to amplify a 162 bp unique fragment in *B. melitensis*. Primer pair 1, which was expected to amplify a unique region in *B. suis 1330*, produced a single band of varying size in every one of the 18 *Brucella *biovars. This polymorphic region encoding for an immunoglobulin-binding protein has a potential diagnostic application.

**Figure 3:**
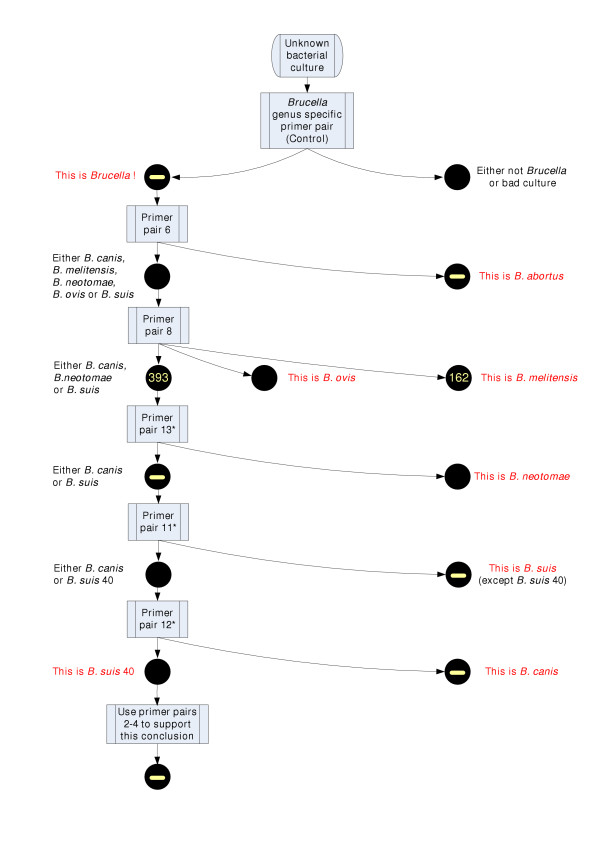
**PCR assay which differentiates between the *Brucella *strains**. PCR assay sequence which differentiates between classical Brucella biovars based on the patterns observed by PCR screening several Brucella biovars. * The primer pair sequences are embedded in the text.

The patterns we observed by PCR screening several *Brucella *biovars can be used in a simple sequence of PCR assays, which differentiates between the classical Brucella strains (Figure 3). The assay starts with an unknown bacterial culture, which is tested with a genus specific primer pair capable to amplify a DNA fragment from any bacterial strain of genus *Brucella*. The primer pair 6 is highly specific to *B. abortus *and amplifies a single band in the seven biovars that were tested. If the primer pair 6 fails to produce a fragment, the bacterial culture that we test belongs to *B. canis, B. melitensis, B. neotomae, B. ovis *or *B. suis*. The PCR primer pair 8 helps to rule out two of the *Brucella *species by giving a substantially shorter fragment in all three *B. melitensis *biovars, and no amplicon in *B. ovis *1155. Primer pair 13 (5'-ACC TCG GCA TGT AAC TCA GG-3' and 5'-ACC CTC CAC ACC AAT AGA CG-3') separates *B. neotomae *5K33. The next step in the diagnostic assay is to separate *B. canis *from *B. suis*. Although computational analysis identified the presence of large unique islands in *Brucella suis *1330, the PCR results revealed that these islands are absent from *B. suis *513 and found in the evolutionary related *B. canis *RM and also *B. neotomae *5K33 biovars. Use of the primer pair 11 (5'-TCG GCC TGT GGA TCT ATT TC-3' and 5'-TTC CAC TTG CGT CAC TGT TC-3') can separate most of the *B. suis *biovars, but an additional PCR with the primer pair 12 (5'-TTG TTG GAA ACG GCT TTG ATA TCC AC-3' and 5'-GAA AGT ACC CAC CCT CGG AAA ACT CC-3') is necessary to separate *B. suis *40 from *B. canis *RM. At every identification step additional PCR reactions may be set up to confirm the *Brucella *species identity. The same differential regions can be used as the discriminatory features on a diagnostic microarray. The primers used in this assay have been screened against all sequences currently present in GenBank. The primer pairs 5, 6, 9 and Control revealed significant full length matches at the nucleotide sequence level only to *Brucella *spp., when compared to the complete GenBank database. Primer pairs designed for these sequences were also found to be unique when compared to the complete GenBank database with BLASTn in short nearly exact match mode. Primer pairs were considered unique if both of the primers in a pair did not have a short nearly exact match hit in the same genome, or, if both did have a short hit in the same genome, the predicted amplicon was longer than 100000 bp or the primer sequence hits were shorter than 14/20 bp. We determined, using Hyther [40-42], that duplexes of 14 nt and below had melting temperatures below the annealing temperature used in the experiment. Primer pairs described as unique to *Brucella *spp. meet these criteria and, therefore, may be useful to verify the presence of *Brucella *specific DNA even in the presence of the host DNA.

## Conclusion

Differentiating genes identified in a comprehensive whole-genome comparison among sequenced *Brucella *biovars have been used successfully as targets to discriminate among *Brucella *strains using a small number of strategically selected PCR assays. The successful differentiating targets have been placed as features on a discriminatory synthetic 70 mer oligonucleotide array for diagnosis of *Brucella *infections, as well as a more comprehensive *Brucella *array that will be used to examine differential gene expression during host-pathogen interactions. With these experiments, we hope to determine whether differences in virulence or host preferences between *Brucella *spp. are due to unique genes or differences in transcription and expression. The information we can obtain from differential expression studies will complement recent research in comparative proteomics of *Brucella *[43,44]. None of the differentiating genes for *B. melitensis *that we identified have yet been detected in the proteome *in vitro*; however, the above proteomics study resulted in annotation of only 6% of the predicted genes in *B. melitensis*. We anticipate that the answers to questions about host preference and virulence will lie in the results obtained from a combination of microarray and functional analyses of mutant strains suggested by genomic analysis and global gene expression approaches.

## Methods

### Genome sequence data and annotation

The *B. abortus *genome has been recently completely sequenced and annotated using Artemis releases 4 and 5 this year [16]. As of today, the complete, annotated genome sequences of *B. abortus *[GenBank:AE017223, GenBank:AE017224], *B. melitensis *[GenBank:AE008917, GenBank:AE008918] and *B. suis *[GenBank:AE014291, GenBank:AE014292] are available in GenBank. The genome of *B. suis *was sequenced at TIGR, and annotated using their standard procedures [14]. *B. melitensis *has been annotated [15] using the ERGO bioinformatics suite. However, at the time this comparison was performed, a complete annotation had not been published for *B. abortus*. Draft *B. abortus *sequence and preliminary annotations were used to represent *B. abortus *in the three-way comparison, along with over 2,000 nucleotide sequence records for *B. abortus *that are available in GenBank. We used annotated coding regions from the published sequences of *B. melitensis *and *B. suis *as the basis for protein-to-protein comparisons.

### Whole genome sequence comparison

Pairwise whole genome alignments for each combination of genomes were performed using MUMmer (v. 2.1) [45]. This analysis facilitates identification of regions of non-identity and single nucleotide polymorphisms between pairs of genomes with high sequence similarity.

### Sequence similarity comparison

Sequence based local alignments were performed using standalone BLAST [46]. A two-stage process using two BLAST programs (tblastx, blastn) was used to define regions of sequence match between genomes. Predicted coding sequences in each genome were translated and compared to each other. Protein sequences were also compared to six-frame-translated genomic sequence to detect homologies that lay outside annotated gene boundaries. In the case of *B. abortus*, no annotation was available at the time of the comparison, and this process was necessary to detect putative gene homologs. Translated genome sequences of *B. melitensis *and *B. suis *were also compared to predicted proteins from other published sequences, in order to detect possible gene homologs which may not have been identified in the published annotations.

BLAST was run for each unique pairing of the three genomes. An e-value cutoff of 0.005 was used for comparisons, and the BLOSUM62 scoring matrix was used for protein-sequence-based comparisons. Genes identified in one genome, for which there were no significant matches either in coding sequence (CDS) or genomic DNA of another, were considered absent in the second genome. We observed in the genomes of these three *Brucella spp*. that either gene homologs existed, and had greater than 90% sequence identity, or that no apparent homolog existed; thus criteria for presence or absence of a gene were simple to establish. Differentiating genes were defined as genes for which we found no significant homology in one or both of the other genomes. A small number of gene pairs identified as matches, but having less than 80% sequence coverage of one or the other homolog, were examined more closely and classified as secondary discriminating features. Partial coverage high-scoring pairs (HSPs) for differentials were examined to determine if they could be combined to make a single match to meet our coverage cutoffs.

As an additional test of uniqueness, primer pairs designed for each of the differentiating sequences were used to query the complete GenBank database in short nearly exact match mode, to identify potential annealing sites not detected in the standard BLASTn search.

### PCR and RT-PCR protocols

*B. suis*, *B. melitensis *and *B. abortus *cultures were grown at 37°C for 36 hours in trypticase soy broth (Difco) and harvested at an OD_550 _= 0.8. The culture was quickly harvested by centrifugation and re-suspended in TE/Citrate/zwittergent 3-14/lysozyme lysing buffer [47]. RNA was extracted using an RNA extraction kit (Qiagen). RNA was quantified by spectrophotometric analysis. Residual genomic DNA contamination was eliminated by treatment with 5 units of DNAse1 (TaKaRa) for 1 hour at room temperature.

Primers were designed using the Primer3 software [48] with a melting temperature of 60°C, G+C content of 50% and primer length of close to 20 bp using default values for the rest of the parameters. Our primers were determined using Nucleic Acid Quikfold (MFold version 3.1 and the SantaLucia free energy parameters for DNA) to have a T_m _of secondary structure formation less than 40°C, and the 2-State Hybridization Server for DNA-DNA-hybrid formation [49] was used to verify duplex melting temperature. An existing set of *B. suis *primer pairs (courtesy of Dr. Ian Paulsen, TIGR), originally designed for a cDNA microarray experiment, contained primers that spanned some of the differential regions and were 100% identical to their target sequences in all three *Brucella *genomes; 22 pairs of primers from this set were used. We also used 81 additional forward or reverse primers from this set, and the primer or primers required to complete the pair in each case were designed by us, based on the *B. melitensis *or *B. suis *annotated genomic sequence as applicable in each case. Additional file 1 contains the sequences of all primer pairs that were used in the RT-PCR and PCR analysis of the differential regions.

Reverse transcription was carried out using the Superscript first-strand synthesis system for RT-PCR (Invitrogen) following prescribed protocols. The synthesized cDNA from each *Brucella *species was used in a PCR reaction as the template with primers specific for each differentiating gene. Ready-to-go PCR beads (puRETaq, Amersham Biosciences) were used according to manufacturer's recommendations. Thermocycling was carried out in the gradient Mastercycler (Eppendorf). Cycling conditions were 90°C for 5 minutes, 90°C for 1 min, for denaturation 55°C for 30 seconds, for annealing 72°C for 1 min extension for 45 cycles and 70°C for 5 minutes of final extension. The RT-PCR products were electrophoretically separated on 1.5 % (TAE/TBE) agarose gels. Primers that were suspected of producing nonspecific bands were retested with a 57°C annealing temperature. When the expected products were longer than 1 kb an increased extension time of 3 minutes was used in the second round of PCR reactions, keeping all other conditions the same.

The genomic DNA for the PCR reactions was extracted using a phenol/chloroform protocol [50]. PCR reactions were performed simultaneously for all three *Brucella *species. The reactions were carried out in a final volume of 30 μl. Sterile water (26 μl) was added to the Amersham Biosciences puReTaq Ready-To-Go-PCR bead (each bead contains 2.5 units of PuReTaq DNA Polymerase) to give: 1.5 mM MgCl_2_, 50 mM KCl, 10 mM Tris-HCl, and 200 μM of each dNTP. The primer and genomic DNA concentrations were 10 pmol and 50 ng respectively. The DNA underwent denaturation for 5 min. at 95°C, followed by 40 cycles consisting of 1 min. of denaturation at 95 °C, 1 min. for primer annealing at 55°C and 3 min. extension time at 72°C, and 72°C for 10 min. of final extension. The PCR products were analyzed by 1% TBE agarose gel electrophoresis.

### PCR screening of Brucella biovars

Eighteen designated type strains of the six classical *Brucella *biovars [51,52] were used to check the applicability of the identified differential regions for the diagnostic testing of *Brucella species*. The *Brucella *cells were obtained from Dr. Betsy J. Bricker at USDA, Ames Iowa, and used to set up the total of over 400 PCR reactions. The cell samples assayed included: *Brucella abortus *(biovars 544, 86/8/59, Tulya, 292, B3196, 870 and C68), *B. canis *RM, *B. melitensis *(biovars 16 M, 63/9 and Ether), *B. neotomae *5K33, *B. ovis *1155 and *B. suis *(biovars 1330, Thompsen, 686, 40 and 513).

Originally, 24 primer pairs were selected to equally represent the unique and differential ORFs we identified. A *Brucella *genus-specific PCR primer pair was designed and used as a positive control for the PCR assay of the differentiating regions. This primer pair was screened against all sequences from all organisms currently deposited in the GenBank Database, and is expected to be extremely *Brucella *specific. Each set of PCR reactions also contained a no DNA contamination control. The PCR was performed on methanol killed bacterial cells, which is a commonly used diagnostic technique [53,54]. The cells were diluted in water down to 0.2-0.15 OD_550 _nm the night before the PCR analysis. The PCR amplification was performed using the PCR SuperMix from Invitrogen, with 55°C primer annealing temperature and 1 minute elongation time. The amplification products were then separated on the 1.6% agarose gel in a sodium borate buffer.

## Authors' contributions

VGR participated in the design of the study, carried out the PCR screening of *Brucella *biovars and drafted the manuscript. DMS performed the computational analysis and drafted the manuscript, SR performed the RT-PCR analysis of differentiating genes. SAR performed the PCR analysis of differentiating genes. YH, RL and NS helped to analyze the data and critically revised the manuscript. SMH contributed the draft genome sequence of *B. abortus *and critically revised the manuscript. SMB and CJG conceived of the study, and participated in its design and coordination and helped to draft the manuscript. All authors read and approved the final manuscript.

## Supplementary Material

Additional file 1 **Primer sequences for amplification of the differential regions in *Brucella***. The additional file is a table in Microsoft *.doc format containing the sequences of all primer pairs that were used in the RT-PCR and PCR analysis of *Brucella *differential regions. Click here for file

## Figures and Tables

**Table 1:** The GenBank Coordinates of observed *Brucella *gene differentials. GenBank coordinates of 102 differential open reading frames identified in the three sequenced *Brucella *species (*B. abortus *9-941, *B. melitensis *16 M and *B. suis *1330) along with their names, sizes and predicted functions.

***B. suis *1330**		***B. melitensis *16 M**	***B. abortus *9-941**		
Chr	Gene Name	Gene Name	Gene Name	Gene Size	Gene Function
1	BR0221 (232958 .. 233407)		BruAb1_0216 (234314 .. 234763)	450	transcriptional regulator, MerR family
1	BR0389 (397661 .. 397933)		BruAb1_0414 (419791 .. 419519)	273	hypothetical protein
1	BR0390 397923 .. 398030		Not annotated 419781 .. 419888	108	hypothetical protein
1	BR0588 581318 .. 581986		BruAb1_0609 602821 .. 603489	669	protease, putative
1	BR0589 582008 .. 583282		BruAb1_0610 603511 .. 604785	1275	major capsid protein, HK97 family
1	BR0590 583447.. 584013		BruAb1_0611 604950 .. 605516	567	conserved hypothetical protein
1	BR0591 584010 .. 584348		BruAb1_0612 605513 .. 605851	339	conserved hypothetical protein
1	BR0592 584345 .. 584512		BruAb1_0613 605848 .. 606381	168/534	hypothetical protein
1	BR0593 584472 .. 584879		BruAb1_0613 605848 .. 606381	408/534	conserved hypothetical protein
1	BR0952 (924995 .. 925828)			834	amino acid ABC transporter, permease protein
1	BR0953 (925831..926553)			723	amino acid ABC transporter, permease protein
1	BR0954 (926569..926748)			180	hypothetical protein
1	BR1060 (1030564 .. 1031664)	BMEI0926 957356 .. 957958		1101/603	multidrug resistance protein A, HlyD family secretion protein
1	BR1057 (1025909 .. 1026703)	BMEI0929 961675 .. 962442		795/767	diguanylate cyclase/phosphodiesterase domain 1 (GGDEF)
1		BMEI0900 933957..934199	BruAb1_1088 (1071663..1072043)	243/380	hypothetical protein
1	BR1846 (1777721 .. 1778557)			837	hypothetical protein
1	BR1852 1782720 .. 1784648		BruAb1_1831 1799556 .. 1801508	1929/1952	transcriptional regulator, Cro/CI family
1	BR1853 1784645 .. 1785319		BruAb1_1832 1801505 .. 1802179	675	AzlC family protein
1		BMEI1661 (1713088 .. 1713834)		747	recombinase
1		BMEI1674 1724077 .. 1724829	BruAb1_0274 (279593 .. 280345)	753	hypothetical protein
1		BMEI1675 (1724950 .. 1725186)	BruAb1_0273 279278 .. 279472	237/194	hypothetical protein
1		BMEI1676 (1725529 .. 1726137)	BruAb1_0271 278285 .. 278893	609	hypothetical protein
1		BMEI1677 (1726408 .. 1726872)	BruAb1_0270 277475 .. 278014	465/539	hypothetical protein
1		BMEI1678 (1726944 .. 1727234)	BruAb1_0269 277188 .. 277478	291	hypothetical protein
1		BMEI1679 (1727300 .. 1727545)	BruAb1_0268 276877 .. 277122	246	hypothetical protein
1		BMEI1680 1727623 .. 1727868	BruAb1_0267 (276554 .. 276799)	246	hypothetical protein
1		BMEI1681 1727932 .. 1728408	BruAb1_0266 (276014 .. 276490)	477	hypothetical protein
1		BMEI1682 1728405 .. 1728890	BruAb1_0265 (275532 .. 276017)	470/486	hypothetical protein
1		BMEI1683 (1729275 .. 1729823)	BruAb1_0264 274599 .. 275147	549	zinc-dependent metallopeptidase
1		BMEI1684 1729845 .. 1730009	BruAb1_0263 274246 .. 274602	165/357	hypothetical protein
1		BMEI1685 1730353 .. 1730586	BruAb1_0262 (273836 .. 274069)	234	hypothetical protein
1		BMEI1686 (1730670 .. 1731023)	BruAb1_0261 273399 .. 273752	354	hypothetical protein
1		BMEI1687 (1731087 .. 1731293)	BruAb1_0260 273129 .. 273335	207	hypothetical protein
1		BMEI1688 (1731290 .. 1731880)	BruAb1_0259 272653 .. 273132	591/479	hypothetical protein
1		BMEI1689 (1731871 .. 1732350)	BruAb1_0258 272071 .. 272550	480	hypothetical protein
1		BMEI1690 (1732392 .. 1732697)	BruAb1_0257 271724 .. 272029	306	hypothetical protein
1		BMEI1691 (1732898 .. 1734790)	BruAb1_0256 269642 .. 271523	1893/1887	hypothetical membrane spanning protein
1		BMEI1692 (1734940 .. 1736856)	BruAb1_0255 267576 .. 269492	1917	flagellar protein FlgJ
1		BMEI1693 (1737057 .. 1737266)	BruAb1_0254 267166 .. 267375	210	hypothetical protein
1		BMEI1694 (1737368 .. 1738384)	BruAb1_0253 266048 .. 267064	1017	hypothetical protein
1		BMEI1695 (1738538 .. 1739155)	BruAb1_0252 265457 .. 265894	618/438	hypothetical protein
1		BMEI1696 (1739125 .. 1740654)	BruAb1_0251 263780 .. 265308	1530/1529	hypothetical membrane spanning protein
1		BMEI1697 (1740651 .. 1741532)	BruAb1_0250 262902..263783	882	virulence-associated protein E
1		BMEI1698 (1741529 .. 1741843)	BruAb1_0249 262591 .. 262905	315	hypothetical protein
1		BMEI1699 (1741840 .. 1742049)	BruAb1_0248 262385 .. 262594	210	hypothetical protein
1		BMEI1700 (1742050 .. 1742268)	BruAb1_0247 262166 .. 262384	219	hypothetical protein
1		BMEI1701 1742369 .. 1742641	BruAb1_0246 261652 .. 262098	262/446	hypothetical protein
1		BMEI1702 (1742779 .. 1743975)	BruAb1_0245 260459 .. 261655	1197	transposase
2	BRA0227 (214967 .. 215674)	BMEII1016 1054886 .. 1055593		708	protease I
2	BRA0362 343695 .. 344903			1209	site-specific recombinase, phage integrase family
2	BRA0363 345188 .. 345418			231	DNA-binding protein, putative
2	BRA0364 345499 .. 346557			1059	RepA-related protein
2	BRA0365 (347606 .. 347932)			327	hypothetical protein
2	BRA0366 (347935 .. 349578)			1644	TrbL protein
2	BRA0367 (349581 .. 349763)			183	hypothetical protein
2	BRA0368 (349766 .. 350557)			792	TrbJ protein
2	BRA0369 350655 .. 350807			153	hypothetical protein
2	BRA0370 (350825 .. 351049)			225	hypothetical protein
2	BRA0371 (351052 .. 351270)			219	TraC protein
2	BRA0372 351940 .. 352320			381	TraJ protein
2	BRA0373 352317 .. 354269			953	TraI protein, putative
2	BRA0374 (354676 .. 356100)			1425	hypothetical protein
2	BRA0375 (356254 .. 357279)			1026	hypothetical protein
2	BRA0376 (357313 .. 358038)			726	hypothetical protein
2	BRA0377 (358217 .. 359938)			1722	conserved hypothetical protein
2	BRA0378 (360180 .. 361004)			825	hypothetical protein
2	BRA0379 (361149 .. 361343)			195	DNA-damage-inducible protein J, putative
2	BRA0418 (402846 .. 403826)	BMEII0849 885138 .. 885878		981/740	GDP-4-dehydro-d-rhamnose reductase
2	BRA0419 (403810 .. 404880)	BMEII0848 884084 .. 885154		1071	GDP-mannose 4,6-dehydratase
2	BRA0420 405144 .. 406394	BMEII0847 (882570 .. 883886)		1250/1317	glycosyltransferase
2	BRA0421 406415 .. 407650	BMEII0846 (881314 .. 882537)		1236/1224	glycosyltransferase, group 1 family protein
2	BRA0422 407647 .. 408843	BMEII0845 (880193 .. 881317)		1197/1125	lipopolysaccharide n-acetylglucosaminyltransferase
2	BRA0423 (408914 .. 409636)	BMEII0844 879463 .. 880122		723/660	outer membrane protein, 31 kDa Found in *B. neotomae*
2	BRA0424 (410033 .. 410647)	BMEII0843 878500 .. 879003		615/504	acetyltransferase, CysE/LacA/LpxA/NodL family
2	BRA0425 (410659 .. 411921)	BMEII0842 877115 .. 878377		1263	hypothetical protein
2	BRA0426 (411918 .. 412535)	BMEII0841 876576 .. 877118		618/543	hypothetical protein
2	BRA0427 (412532 .. 413413)	BMEII0840 875548 .. 876504		882/957	glycosyltransferase involved in cell wall biogenesis
2	BRA0428 (413410 .. 414537)	BMEII0839 874562 .. 875626		1128/1065	undecaprenyl-phosphate α-n-acetylglucosaminyl transferase
2	BRA0429 414830 .. 416323	BMEII0838 (872719 .. 874224)		1494/1506	succinoglycan biosynthesis transport protein exot
2	BRA0430 416339 .. 417352	BMEII0837 (871690 .. 872703)		1014	glycosyltransferase, group 2 family protein
2	BRA0431 (417308 .. 418549)	BMEII0836 870493 .. 871734		1242	dTDP-4-dehydrorhamnose 3,5-epimerase
2	BRA0432 418666 .. 420045	BMEII0835 (868997 .. 869920)		1380/924	glycosyltransferase, group 1 family protein
2	BRA0433 420083 .. 421444	BMEII0834 (867598 .. 868959)		1362	glutamate-1-semialdehyde 2,1-aminomutase
2	BRA0434 421423 .. 422757	BMEII0833 (866285 .. 867619)		1335	conserved hypothetical protein
2	BRA0435 422878 .. 423939	BMEII0832 (865103 .. 866170)		1062/1068	UDP-glucose 4-epimerase
2	BRA0436 423939 .. 425291	BMEII0831 (863751 .. 865064)		1353/1314	conserved hypothetical protein
2	BRA0437 (425254 .. 425778)	BMEII0830 863234 .. 863788		525/555	dTDP-4-dehydrorhamnose 3,5-epimerase
2	BRA0438 426099 .. 427400	BMEII0829 (862282 .. 862944)		1302/663	methyltransferase, putative
2	BRA0438 426099 .. 427400	BMEII0828 (861644 .. 862210)		1302/567	possible S-adenosylmethionine-dependent methyltransferase
2	BRA0439 427403 .. 428212	BMEII0827 (860832 .. 861719)		480	glucose-1-phosphate cytidylyltransferase
2	BRA0541 (521842 .. 522066)		BruAb2_0681 692179 .. 692403	225	hypothetical protein
2	BRA0630 (610688 .. 611938)		BruAb2_0596 605152 .. 606402	1251	amino acid dehydrogenase, putative
2	BRA0631 (612027 .. 612788)		BruAb2_0595 604302 .. 605063	762	amino acid ABC transporter
2	BRA0632 (612944 .. 613717)		BruAb2_0594 603373 .. 604146	774	amino acid ABC transporter,
2	BRA0633 (613902 .. 615005)		BruAb2_0593 602085 .. 603188	1104	conserved hypothetical protein
2	BRA0634 615107 .. 615556		BruAb2_0592 (601534 .. 601982)	450	transcriptional regulator, AsnC family
2	BRA0635 615836 .. 617563		BruAb2_0591 (599527 .. 601254)	1728	twin-arginine translocation signal domain protein
2	BRA0636 (617674 .. 618876)		BruAb2_0590 598292 .. 599416	1203/1125	beta-ketoadipyl CoA thiolase
2	BRA0749 731323 .. 732192		BruAb2_0483 (484959 .. 485828)	870	sugar ABC transporter, permease protein, putative
2	BRA0907 888804 .. 890204		BruAb2_0326 (326911 .. 328311)	1401	conserved hypothetical protein
2	BRA1096 1082617 .. 1083330		BruAb2_1035 1037839 .. 1038552	714	transcriptional regulator, putative
2	BRA0553 (532630 .. 534594)	BMEII0717 755374 .. 757398		1965/2025	hemagglutinin, cell wall surface protein, putative

**Table 2:** Summary of RT-PCR results from differentiating CDSs *Brucella *species, grouped by differentiating island. Transcripts detected in each differentiating sequence island of *B. abortus *9-941, *B. melitensis *16 M and *B. suis *1330, when the cell cultures were grown at 37°C for 36 hours in trypticase soy broth (Difco).

	***B. suis***			***B. melitensis***			***B. abortus***
	
**Location**	**Predicted**	**Observed**	**No Band**	**Predicted**	**Observed**	**No Band**	**Observed**
***B. suis *Chr. I (S1)**	4	4	-	1	1	-	-
***B. suis *Chr. II (S2)**	18	17	1	-	-	-	-
***B. melitensis *Chr. I (M1)**	-	-	-	1	1	-	-
***B. abortus *Chr. I (A1)**	-	-	-	-	-	-	1
***B. suis *+ *B. melitensis *Chr. I (SM1)**	1	1	-	2	2	-	-
**B. *suis *+ *B. melitensis *Chr. II (SM2)**	25	16	9	24	6	18	-
***B. suis + B. abortus *Chr. I (SA1)**	11	3	8	-	-	-	7
***B. suis *+ *B. abortus *Chr. II (SA2)**	11	7	4	-	-	-	6
***B. melitensis + B. abortus *Chr. I (MA1)**	-	-	-	30	21	9	23

**Table 3:** Detailed results for RT-PCR analysis of differentiating CDSs from *Brucella *species, by gene. Detailed breakdown of the transcripts detected in each differentiating sequence island of *B. abortus *9-941 *, B. melitensis *16 M and *B. suis *1330, when the cell cultures were grown at 37°C for 36 hours in trypticase soy broth (Difco).

				***B. suis***		***B. melitensis***		***B. abortus***	
**#**	**CDS Name**	**Function**	**Amplicon Size (bp)**	**Predicted**	**Observed**	**Predicted**	**Observed**	**Predicted**	**Observed**
A.	*B. suis Chromosome I *(S1)								
1	BR0952	putative amino acid ABC transporter, permease protein	**396**	**+**	**+**	**-**	**-**	**-**	**-**
2	BR0953	putative amino acid ABC transporter, permease protein	**438**	**+**	**+**	**-**	**-**	**-**	**-**
3	BR0954	hypothetical protein	**153**	**+**	**+**	**-**	**-**	**-**	**-**
4	BR1846	hypothetical protein	**721^S^** **469^M^**	**+**	**+**	**+**	**+**	**-**	**-**
									
B.	*B. suis Chromosome II *(S2)								

5	BRA0362	putative site-specific recombinase, phage integrase family	**722**	**+**	**+**	**-**	**-**	**-**	**-**
6	BRA0363	putative DNA-binding protein	**148**	**+**	**+**	**-**	**-**	**-**	**-**
7	BRA0364	putative RepA-related protein	**655**	**+**	**+**	**-**	**-**	**-**	**-**
8	BRA0365	hypothetical protein	**167**	**+**	**+**	**-**	**-**	**-**	**-**
9	BRA0366	putative TrbL protein	**170**	**+**	**+**	**-**	**-**	**-**	**-**
10	BRA0367	putative TrbL protein	**119**	**+**	**+**	**-**	**-**	**-**	**-**
11	BRA0368	putative TrbJ protein	**354**	**+**	**+**	**-**	**-**	**-**	**-**
12	BRA0369	hypothetical protein	**123**	**+**	**+**	**-**	**-**	**-**	**-**
13	BRA0370	hypothetical protein	**121**	**+**	**+**	**-**	**-**	**-**	**-**
14	BRA0371	putative TraC protein	**140**	**+**	**+**	**-**	**-**	**-**	**-**
15	BRA0372	putative TraJ protein	**218**	**+**	**+**	**-**	**-**	**-**	**-**
16	BRA0373	putative TraI protein	**173**	**+**	**-**	**-**	**-**	**-**	**-**
17	BRA0374	hypothetical protein	**768**	**+**	**+**	**-**	**-**	**-**	**-**
18	BRA0375	hypothetical protein	**648**	**+**	**+**	**-**	**-**	**-**	**-**
19	BRA0376	hypothetical protein	**532**	**+**	**+**	**-**	**-**	**-**	**-**
20	BRA0377	conserved hypothetical protein	**867**	**+**	**+**	**-**	**-**	**-**	**-**
21	BRA0378	hypothetical protein	**191**	**+**	**+**	**-**	**-**	**-**	**-**
22	BRA0379	putative DNA-damage-inducible protein J	**119**	**+**	**+**	**-**	**-**	**-**	**-**
									
C.	*B. melitensis Chromosome I *(M1)								

23	BMEI1661	recombinase	**218**	**-**	**-**	**+**	**+**	**-**	**-**
									
D.	*B. abortus*								

24	6 kb Partial differential, primer pair 1		**782**	**-**	-	-	-	+	+
25	6 kb Partial differential, primer pair 2		**613^M^** **1142^S^** **4484^A^**	**+**	-	+	-	+	-
									
E.	*B. suis and B. melitensis Chromosome I *(SM1)								

26	BR1060/BMEI0926/	putative HlyD family secretion protein/multidrug resistance protein A	**207**	**-**	**-**	**+**	**+**	**-**	**-**
27	BR1057/BMEI0929	Diguanylate cyclase/phosphodiesterase domain/putative GGDEF domain protein	**323**	**+**	**+**	**+**	**+**	**-**	**-**
									
F.	*B. suis and B. melitensis Chromosome II *(SM2)								

28	BRA0227/BMEII1016	putative ThiJ/PfpI family protein/protease I	**466**	+	+	+	-	-	-
29	BRA0418/BMEII0849	putative fucose synthetase family protein/GDP-4-dehydro-D-rhamnose reductase	**363**	**+**	**+**	**+**	**-**	**-**	**-**
30	BRA0419/BMEII0848	putative GDP-mannose 4,6-dehydratase Bme9/GDP-mannose 4,6-dehydratase	**239**	**+**	**-**	**+**	**+**	**-**	**-**
31	BRA0420/BMEII0847	putative glycosyltransferase/glycosyl transferase	**657**	**+**	**+**	**+**	**-**	**-**	**-**
32	BRA0421/BMEII0846	putative glycosyltransferase, group 1 family protein/glycosyl transferase	**229**	**+**	**-**	**+**	**-**	**-**	**-**
33	BRA0422/BMEII0845	putative glycosyltransferase, group 1 family protein/: lipopolysaccharide N-acetylglucosaminyltransferase	**470^S^** **398^M^**	**+**	**+**	**+**	**-**	**-**	**-**
34	BRA0423/BMEII0844	putative outer membrane protein, 31 kDa/31 kDa outer-membrane immunogenic protein precursor	**317**	**+**	**+**	**+**	**+**	**-**	**-**
35	BRA0424/BMEII0843	putative acetyltransferase, CysE/LacA/LpxA/NodL family/putative colanic acid biosynthesis acetyltransferase WCAF	**366**	**+**	**-**	**+**	**-**	**-**	**-**
36	BRA0425/BMEII0842	putative membrane protein Bme3/hypothetical protein	**774**	**+**	**-**	**+**	**-**	**-**	**-**
37	BRA0426/BMEII0841	putative Bme2 protein/hypothetical protein	**286**	**+**	**-**	**+**	**+**	**-**	**-**
38	BRA0427/BMEII0840	putative glycosyl transferase, group 2 family protein/glycosyltransferase involved in cell wall biogenesis	**279**	**+**	**+**	**-**	**-**	**-**	**-**
39	BRA0428/BMEII0839	putative undecaprenyl-phosphate alpha-N-acetylglucosaminyltransferase	**672**	**+**	**+**	**+**	**-**	**-**	**-**
40	BRA0429/BMEII0838	putative polysaccharide biosynthesis protein/succinoglycan biosynthesis transport protein exot	**306**	**+**	**-**	**+**	**-**	**-**	**-**
41	BRA0430/BMEII0837	putative glycosyltransferase, group 2 family protein/glycosyltransferase	**488**	**+**	**-**	**+**	**-**	**-**	**-**
42	BRA0431/BMEII0836	conserved hypothetical protein/dTDP-4-dehydrorhamnose 3,5-epimerase	**281**	**+**	**-**	**+**	**-**	**-**	**-**
43	BRA0432/BMEII0835	putative glycosyltransferase, group 1 family protein/glycosyltransferase	**223^S^** **708^M^**	**+**	**+**	**+**	**+**	**-**	**-**
44	BRA0433/BMEII0834	putative glutamate-1-semialdehyde-2,1-aminomutase/glutamate-1-semialdehyde 2,1-aminomutase	**463**	**+**	**+**	**+**	**-**	**-**	**-**
45	BRA0434/BMEII0833	putative conserved hypothetical protein/hypothetical protein	**239**	**+**	**+**	**+**	**-**	**-**	**-**
46	BRA0435/BMEII0832	putative epimerase/dehydratase family protein/UDP-glucose 4-epimerase	**642**	**+**	**+**	**+**	**-**	**-**	**-**
47	BRA0436/BMEII0831	conserved hypothetical protein/hypothetical protein	**188**	**+**	**+**	**+**	**+**	**-**	**-**
48	BRA0437/BMEII0830	putative dTDP-4-dehydrorhamnose 3,5-epimerase/dTDP-4-dehydrorhamnose 3,5-epimerase dTDP-4-dehydrorhamnose reductase	**285**	**+**	**+**	**+**	**-**	**-**	**-**
49	BRA0438/BMEII0828	putative methyltransferase/possible s-adenosylmethionine-dependent methyltransferase	**452**	**+**	**+**	**+**	**-**	**-**	**-**
50	BRA0438/BMEII0829	putative methyltransferase/possible s-adenosylmethionine-dependent methyltransferase	**155**	**+**	**-**	**+**	**-**	**-**	**-**
51	BRA0439/BMEII0827	putative nucleotidyltransferase family protein/glucose-1-phosphate cytidylyltransferase	**525**	**+**	**+**	**+**	**+**	**-**	**-**
52	BRA0553/BMEII0717	putative cell wall surface protein/hemagglutinin	**421**	**+**	**+**	**+**	**-**	**-**	**-**
									
G.	*B. suis and B. abortus Chromosome I *(SA1)								

53	BR0221/BruAb1_0216	putative transcriptional regulator, MerR family	**91**	**+**	**+**	**-**	**-**	**+**	**+**
54	BR0389/BruAb1_0414	hypothetical protein	**141**	**+**	**-**	**-**	**-**	**+**	**+**
55	BR0390/Not annotated	hypothetical protein	**74**	**+**	**-**	**-**	**-**	**-**	**-**
56	BR0588/BruAb1_0609	putative protease	**665**	**+**	**-**	**-**	**-**	**+**	**+**
57	BR0589/BruAb1_0610	major capsid protein, HK97 family/putative protein	**303**	**+**	**-**	**-**	**-**	**+**	**+**
58	BR0590/BruAb1_0611	conserved hypothetical protein	**71**	**+**	**-**	**-**	**-**	**+**	**+**
59	BR0591/BruAb1_0612	conserved hypothetical protein	**139**	**+**	**-**	**-**	**-**	**+**	**-**
60	BR0592 BruAb1_0613	hypothetical protein	**91**	**+**	**-**	**-**	**-**	**+**	**-**
61	BR0593/BruAb1_0613	conserved hypothetical protein	**208**	**+**	**+**	**-**	**-**	**+**	**-**
62	BR1852/BruAb1_1831	transcriptional regulator, Cro/CI family,	**194**	**+**	**-**	**-**	**-**	**+**	**+**
63	BR1853/BruAb1_1832	putative AzlC family protein	**610**	**+**	**+**	**-**	**-**	**+**	**+**
									
H.	*B. suis and B. abortus Chromosome II *(SA2)								

64	BRA0541/BruAb2_0681	hypothetical protein	**118**	**+**	**-**	**-**	**-**	**+**	**-**
65	BRA0630/BruAb2_0596	putative amino acid dehydrogenase	**736**	**+**	**+**	**-**	**-**	**+**	**+**
66	BRA0631/BruAb2_0595	putative amino acid ABC transporter, periplasmic amino acid-binding protein	**202**	**+**	**-**	**-**	**-**	**+**	**-**
67	BRA0632/BruAb2_0594	putative amino acid ABC transporter, periplasmic amino acid-binding protein	**321**	**+**	**+**	**-**	**-**	**+**	**-**
68	BRA0633/BruAb2_0593	conserved hypothetical protein	**591**	**+**	**+**	**-**	**-**	**+**	**+**
69	BRA0634/BruAb2_0592	putative transcriptional regulator, AsnC family	**276**	**+**	**-**	**-**	**-**	**+**	**-**
70	BRA0635/BruAb2_0591	putative twin-arginine translocation signal domain protein	**998**	**+**	**+**	**-**	**-**	**+**	**+**
71	BRA0636/BruAb2_0590	putative beta-ketoadipyl CoA thiolase	**635**	**+**	**+**	**-**	**-**	**+**	**-**
72	BRA0749/BruAb2_0483	putative sugar ABC transporter, permease protein	**310**	**+**	**+**	**-**	**-**	**+**	**+**
73	BRA0907/BruAb2_0326	conserved hypothetical protein	**825**	**+**	**-**	**-**	**-**	**+**	**+**
74	BRA1096/BruAb2_1035	putative transcriptional regulator	**393**	**+**	**+**	**-**	**-**	**+**	**+**
									
I.	*B. melitensis and B. abortus Chromosome I *(MA1)								

75	BMEI0900/BruAb1_1088	hypothetical protein	**212**	**-**	**-**	**+**	**+**	**+**	**-**
76	BMEI1674/BruAb1_0274	hypothetical protein	**597**	**-**	**-**	**+**	**+**	**+**	**+**
77	BMEI1675 BruAb1_0273	hypothetical protein	**157**	**-**	**-**	**+**	**+**	**+**	**+**
78	BMEI1676/BruAb1_0271	hypothetical protein	**206**	**-**	**-**	**+**	**-**	**+**	**+**
79	BMEI1977/BruAb1_0270	hypothetical protein	**400**	**-**	**-**	**+**	**+**	**+**	**+**
80	BMEI1978/BruAb1_0269	hypothetical protein	**192**	**-**	**-**	**+**	**+**	**+**	**+**
81	BMEI1979/BruAb1_0268	hypothetical protein	**201**	**-**	**-**	**+**	**+**	**+**	**+**
82	BMEI1980/BruAb1_0267	hypothetical protein	**210**	**-**	**-**	**+**	**+**	**+**	**+**
83	BMEI1981/BruAb1_0266	hypothetical protein	**358**	**-**	**-**	**+**	**+**	**+**	**+**
84	BMEI1982/BruAb1_0265	hypothetical protein	**418**	**-**	**-**	**+**	**+**	**+**	**+**
85	BMEI1683/BruAb1_0264	zinc-dependent metallopeptidase	**482**	**-**	**-**	**+**	**+**	**+**	**+**
86	BMEI1684/BruAb1_0263	hypothetical protein	**149**	**-**	**-**	**+**	**-**	**+**	**-**
87	BMEI1685/BruAb1_0262	hypothetical protein	**163**	**-**	**-**	**+**	**-**	**+**	**-**
88	BMEI1686/BruAb1_0261	hypothetical protein	**265**	**-**	**-**	**+**	**+**	**+**	**+**
89	BMEI1687/BruAb1_0260	hypothetical protein	**167**	**-**	**-**	**+**	**+**	**+**	**+**
90	BMEI1688/BruAb1_0259	hypothetical protein	**431**	**-**	**-**	**+**	**-**	**+**	**-**
91	BMEI1689/BruAb1_0258	hypothetical protein	**271**	**-**	**-**	**+**	**+**	**+**	**+**
92	BMEI1690/BruAb1_0257	hypothetical protein	**160**	**-**	**-**	**+**	**-**	**+**	**+**
93	BMEI1691/BruAb1_0256	hypothetical membrane spanning protein	**206**	**-**	**-**	**+**	**-**	**+**	**-**
94	BMEI1692/BruAb1_0255	flagellar protein FlgJ	**201**	**-**	**-**	**+**	**-**	**+**	**-**
95	BMEI1693/BruAb1_0254	hypothetical protein	**151**	**-**	**-**	**+**	**+**	**+**	**+**
96	BMEI1694/BruAb1_0253	hypothetical protein	**150**	**-**	**-**	**+**	**-**	**+**	**+**
97	BMEI1695/BruAb1_0252	hypothetical protein	**239**	**-**	**-**	**+**	**+**	**+**	**+**
98	BMEI1696/BruAb1_0251	hypothetical membrane spanning protein	**526**	**-**	**-**	**+**	**+**	**+**	**+**
99	BMEI1697/BruAb1_0250	virulence-associated protein E	**857**	**-**	**-**	**+**	**+**	**+**	**+**
100	BMEI1698/BruAb1_0249	hypothetical protein	**245**	**-**	**-**	**+**	**+**	**+**	**+**
101	BMEI1699/BruAb1_0248	hypothetical protein	**183**	**-**	**-**	**+**	**+**	**+**	**+**
102	BMEI1700/BruAb1_0247	hypothetical protein	**207**	**-**	**-**	**+**	**+**	**+**	**+**
103	BMEI1701/BruAb1_0246	hypothetical protein	**221**	**-**	**-**	**+**	**+**	**+**	**+**
104	BMEI1702/BruAb1_0245	transposase	**169**	**-**	**-**	**+**	**-**	**+**	**-**

+ Obtained RT-PCR fragment of the expected length

- No band was observed in the RT-PCR experiment

^S^size of PCR fragment applies to *B. suis*. ^M^size of PCR fragment applies to *B. melitensis*. ^A^size of PCR fragment applies to *B. abortus*

**Table 4:** Primer pairs used for PCR amplification of unique and differential regions in 18 *Brucella *biovars. Sequences of primer pairs, which where use to PCR amplify several differential genes across the 18 *Brucella *biovars along with the expected amplicon size and predicted gene function.

**Primer pair number**	**Forward primer ... Reverse primer**	**ORF name Amplicon size, bp**			**Gene function**
		
		***B. abortus***	***B. melitensis***	***B. suis***	
**1**	TGATAGCGCCAGACAACAAC **... **TGTGCCAGCTTCGTTGTAAG	BruAb1_1825 596	BMEI0205 470	BR1846 722	Immunoglobulin-binding protein EIBE
**2**	AAATGTCAATCTGGGCTTCG **... **TATTGAAGAACTGCGCAACG			BRA0378 191	Hypothetical protein
**3**	ATTTATGTCCGTGAACTGTCCGTC **... **TTGTCCGCAAAAAGTATCAAAACG			BRA0369 123	Hypothetical protein
**4**	AACTGCTGGAGATGAATCCG **... **GAATGTTTGCACGCATCAAT			BRA0363 149	DNA-binding protein
**5**	CTTTACGCCCGTGTATCGAC **... **CATGGGGTCCTGTGTTGAG		BMEI1661 321		Recombinase
**6**	TGCAGCTCACGGATAATTTG **... **ACACCTTGTCCACGCTCAC	BruAb2_0168 783			Outermembrane transporter
**7**	AGCTTCTGGAGGAGGTGGAT **... **GTTCCGCCTTGTGTTTCTTC		BMEII0827 526	BRA0439 526	Glucose-1-phosphate cytidylyltransferase
**8**	TCTACACCACGCTGAAGTCG **... **CCGAAAGCCGATAGAGTTTG	BruAb2_1035 393	BMEII0204 162	BRA1096 393	Transcriptional regulator, GNTR family
**9**	TTGTTGGAAACGGCTTTGATATC **... **GAAAGTACCCACCCTCGGAAAACT	BruAb1_0266 358	BMEI1681 358		Hypothetical protein
**10**	TCATGCTGTGCCTCCAATTCC **... **TTGCTGAGCAGCAGCAAGAAC	BruAb1_0248 184	BMEI1699 184		Hypothetical protein
**Control**	TCAGGCGCTTATAACCGAAG **... **ATCTGCGCATAGGTCTGCTT	BruAb2_0582 261	BMEII0637 261	BRA0644 261	pcaC 4-carboxymuconolactone decarboxylase
