# Supplementary material for: Molecular targets for rapid identification of Brucella spp
Source: BMC Microbiol. 2006 Feb 22;6:13. doi: 10.1186/1471-2180-6-13 (PMC1413539; doi:10.1186/1471-2180-6-13)
Supplement: Additional file 1 — Primer sequences for amplification of the differential regions in Brucella. The additional file is a table in Microsoft *.doc format containing the sequences of all primer pairs that were used in the RT-PCR and PCR analysis of Brucella differential regions. [file 1471-2180-6-13-S1.doc]

**Additional Table**: Primers used in RT-PCR for proposed differential ORFs from *Brucella* spp***.***

**# ORF Name Amplicon Size (bp) Primer Sequence 1 Primer Sequence 2**

***B. suis Chromosome I***

1 BR0952 **396** CCTTGCTTTCGGCTATGTCT GTTTCGCCCATCAGATCAAG

2 BR0953 **438** CGTTGGTCTCATCCTTGGTT ACCGAAACAAGCGACGTATC

3 BR0954 **153** CAGGCCGTATGTCTAATTCCA GAGCGGTTCCAGTTTGAATG

4 BR1846 **722 in *B. suis* /** TGATAGCGCCAGACAACAAC TGTGCCAGCTTCGTTGTAAG

**469 in *B. melitensis***

***B. suis Chromosome II***

5 BRA0362 **722** AACAAGGTAGGCCATCAACG TGTCCACTCGTCGATCATGT

6 BRA0363 **148** AACTGCTGGAGATGAATCCG GAATGTTTGCACGCATCAAT

7 BRA0364 **655** GAACAGATGGAAAGGGCAAA CCGGCTCGATCAGAAAGTAG

8 BRA0365 **167** GTATTGGTATGTGGGCGCTT TGGCATGCGATTAAGCATAC

9 BRA0366 **170** GTGTTCGTGACGCTCGTAGA TGCATGATCAGACCCACAAT

10 BRA0367 **119** CGCCCTCTGTCTCGTCTC TGCGGAATTGGTCATCCT

11 BRA0368 **354** CGGTGAATACGCAACTGAAC TCTCATCCTCGAATGTGCTG

12 BRA0369 **123** ATTTATGTCCGTGAACTGTCCGTC TTGTCCGCAAAAAGTATCAAAACG

13 BRA0370 **121** GGCCTAGCAAGAGCGTTATG TGTACGCGATAGAGGTCACG

14 BRA0371 **140** GGCTGGCAAAACTTGACAAT ATTCCCCTTGCTGATCCTTT

15 BRA0372 **218** AAAGAGTTAGCCGAAAGGGC ATCGCTTCTACGTCAATCGG

16 BRA0373 **173** GTTCAAACAGACGACGATGC TGTCGAGAACAACAGCCTTG

17 BRA0374 **768** TCCACACAAAAATCCGTTCA CGCTCTACCAAACCTTGCTC

18 BRA0375 **648** GCCACCTACGTCGCTCTTAG AAAGACCCGGTTGCAGTATG

19 BRA0376 **532** TATCGGTTCGATGAGCGATT GCAGGAGATGGTTCTTGAGC

20 BRA0377 **867** ACTTGGCCAATGCTAACACC GGTCATTAAGGTCGAAGCCA

21 BRA0378 **191** AAATGTCAATCTGGGCTTCG TATTGAAGAACTGCGCAACG

22 BRA0379 **119** GGAAGCCACGAACGTGTTAT GAGCCTGCACACGATTGATA

***B. melitensis Chromosome I***

23 BMEI1661 **218** GTGACCGATCAAGGCGTATC GAGCCGTCAAAGGTCATGTT

***B. abortus***

24 6 kb Partial differential, **782** TGCAGCTCACGGATAATTTG ACACCTTGTCCACGCTCAC

primer pair 1

25 6 kb Partial differential, **4,484 in *B. abortus* /** ATCTGGTTCTTTCGGGTGTG CATCACCAAGAACCGTGTTG

primer pair 2 **1142 in *B. melitensis* /**

**613 in *B. suis***

***B. suis and B. melitensis Chromosome I***

26 BR1060 / BMEI0926 **207** GTACATCGGCGACCAAAGAT GGAATCATCCTCGCTCACAT

27 BR1057 / BMEI0929 **323** AAGTCTGAGCCTGGGGTTTC TTACATTTTGCAAGGCGAAG

***B. suis and B. melitensis Chromosome II***

28 BRA0227/ BMEII1016 **466** ATGTGGCCCATTATGCAGAT TCTTCCATCCATGAAAAGCC

29 BRA0418 / BMEII0849 **363** ACAGGCCCGATGTCATTATC GATCGAAATTGTCGTTCGGT

30 BRA0419 / BMEII0848 **239** CAGCTCTCATCGTCGGTGTA AATCTCGTCAGGCTGCACTT

31 BRA0420 / BMEII0847 **657** CGTTACTTCTACCCGGACCA ATAGCCGATGACGAATTTGC

32 BRA0421 / BMEII0846 **229** CAGGCTTTCCAACCAGACT TTCCTCAAGAGCCAGTTGAT

33 BRA0422 / BMEII0845 **470 in *B. suis /*** TATGACCCTGCTAAGGGTGG TCGATTTCATCACGGTTGAG

**398 in *B. melitensis***

34 BRA0423 / BMEII0844 **317** GCTTTGACAAGGAAGACAACG CAGGTTGAACGCAGACTTGA

35 BRA0424 / BMEII0843 **366** GAGCCGATATCCACAAGAGC TTGCTGGATTGCCTGTGTAA

36 BRA0425 / BMEII0842 **774** TATCAGTTCCCGTTTCTGGC GTTCCAGTGAAATTTGCCGT

37 BRA0426 / BMEII0841 **286** ACGGCAAATTTCACTGGAAC GCAGGAACGATGGAGAACA

38 BRA0427 / BMEII0840  **279** TGATGAAATGCTGGCGATAG ACGGAGGAATAACGGTTGTG

39 BRA0428 / BMEII0839 **672** CTTTCTATTGCTTTTCGCCG AAGGTGATGTTCGGTTTTGC

40 BRA0429 / BMEII0838 **306** CCTCTCCAATTTCATTTCGC CAGCACAGGACCGAGACAAT

41 BRA0430 / BMEII0837 **488** AATTATGCCCGTTATCTGCG TTCTGTGCCGATGTTCTGAC

42 BRA0431 / BMEII0836 **281** CAGGGGCTTGATCTCATCAT ACAGTGTCGATGCGGGTATT

43 BRA0432 / BMEII0835  **708** CGACATCATAGATCGCATGG ACGATAGTATCCGTCACGCC

**223** ATCAAGCTGGCTCCCTTTG GAAAACCGTCTTCACCATCG

44 BRA0433 / BMEII0834 **463** GCCTATACAGGGCGTGACAT GGAAATCCGTATGGTGAAGC

45 BRA0434 / BMEII0833 **239** CTTCAACGCTGTTCTGGTCA CCATCTCCATCGCCATATAC

46 BRA0435 / BMEII0832 **642** GATATCGGCCTTTACGAGCA ACGGTAGTTATGCGCGGTAG

47 BRA0436 / BMEII0831 **188** CGTGAAGCCTGTATTGCAGA CTTTCGCCATGATAGCAGGT

48 BRA0437 / BMEII0830 **285** AAACACACTTTCCCCAGCAC TCGAGATCATGTAGCGGATG

49 BRA0438 / BMEII0828 **452** TGAAGGTGATCGATGTGGAA CCGGATAATCTCGTCCTTGA

50 BRA0438 / BMEII0829 **155** TCCGATCCTTGGCATAGAAC CCAGAACATTATTGCCGATG

51 BRA0439 / BMEII0827 **525** AGCTTCTGGAGGAGGTGGAT GTTCCGCCTTGTGTTTCTTC

52 BRA0553 / BMEII0717 **421** AGATAAACATTGCAACCGGC TGACCACCGATCACAATCTG

***B .suis and B. abortus Chromosome I***

53 BR0221 / DI064 **91** AGGAACTGCTGGCGCTCT TTTGCTTCCAGTTCCTCGAT

54 BR0389 **141** GGCGACCAGAAGGATTAGAA TGCATGAAAGCCTTGTTCTG

55 BR0390 / DI073  **74** GTGGCAAACATGCCATCC TATGAGCCGGATGTTTCCT

56 BR0588 **665** GGCGAGATTTGATCTGAAGC AGATCATTGTTCTGGCGACC

57 BR0589 / DI066 **303** GTCCTTGTCCTTCAGCTTGC AGTGGATTGCGGAAGAAGTG

58 BR0590 / DI067 **71** ATTGCAGGCTTTGGTGAGAG GCGGTCAGGGTAAGGATTG

59 BR0591 / DI068 **139** GGAAACATGGTCGGAAATTG ATCGGTCGAAATGTCCTCAC

60 BR0592 **91** TGACCTTTGAAAGCCTCGTT TCCTTGACATTCGGATTTTG

61 BR0593 / DI069 **208** CAGATAGCTGCCAAGACCGT GAGTACGGCCAGTGAAGAGG

62 BR1852 / DI071 **194** CCGCTATTGCGACGTTCTAT AAGCGCTGCCTCTATGATGT

63 BR1853 / DI072 **610** GGGCAAATAGCCAAGAATGA CTTGCCAATAATGCAGCAGA

***B. suis and B. abortus Chromosome II***

64 BRA0541 / DII007 **118** TGGCACTTATGTTCTGGCTG AATCATTGCGAACTCGATCC

65 BRA0630 / DII008 **736** TCCAGCGTCATTCCCTATTC CATAGCCTGCTTCTTCGTCC

66 BRA0631 / DII001  **202** AAGAAGGAAGTCGCGTTCAC GGCAGTCGGGTAAAGTTTCA

67 BRA0632 / DII002 **321** TCAGAACCCGGAATATCACC GCCTTCACCGACCGTTATTA

68 BRA0633 / DII003 **591** CAAGGAACTGGCGAAAGAAG ATCTGCGCAGCATAGACCTT

69 BRA0634 / DII005 **276** ATCGAAGGTTATACGGTGCG GCGTGTGGAAAGGATGATG

70 BRA0635 / DII006 **998** GCTTTTGTTCCCTTTGTGGA TCGCTAACCTTGATCTGGCT

71 BRA0636 / DII009 **635** GAAAACGTTGCCGAGGACTA TTGCCATTGTTAAGCGACAG

72 BRA0749 / DII010 **310** CGTCGCCATCATTCTCAATA TGAGGCTTCGTAGAGGTCGT

73 BRA0907 / DII011 **825** CTTTGTTGCTGAAGTGCTGG TGAAGGGCTTCAATGGATTC

74 BRA1096 / DII012 **393** TCTACACCACGCTGAAGTCG CCGAAAGCCGATAGAGTTTG

***B. melitensis and B. abortus Chromosome I***

75 BMEI0900 **212** GGCCTTTACAAGCACACCAG CTGGAACTCTTTGGCAATCG

76 BMEI1674 / DI002 **597** CCACACAGAAAAGCAAAATGTCAGAGC GACTGAAACGCCGAACTTCATCGTAG

77 BMEI1675 **157** TTCCCATGTTCGATAAGCTGACAAGC TCCTCACAATACGGGTGTCCATTTTG

78 BMEI1676 / DI006 **206** CGTCTATGCGTCCCTCGTAACTATCG GATGTGGGTTCTCGATCCGGTAGAC

79 BMEI1977 / DI008 **400** CGGCTTTGCAGGACCATACATTTATC AGATAGATCAGTGCAGCGCGAAGTG

80 BMEI1978 / DI010 **192** CAAGCAGAAAAAGGAAACCCTGCTTG TCCAGCACCATCTCATTTACCGTTTC

81 BMEI1979 **201** TCATGAATGAAGCATGGAGCACCTAC CTAAAAGGCGATTAATCTGCGCATCG

82 BMEI1980 **210** GCACTTCTCAAAATGGAGCAACGAG CTATCCAACCTTCAACGCCACACC

83 BMEI1981 / DI014 **358** TTGTTGGAAACGGCTTTGATATCCAC GAAAGTACCCACCCTCGGAAAACTCC

84 BMEI1982 / DI015 **418** TTTTATATACATGGGCGCCGCCAAAC GGCTTGTCGATGGGTTTATTTCAAATTC

85 BMEI1683 / DI018 **482** CAGAATTACGTTGTCCCACCGCTTTC GCGCCACGAGATACACTATGTCTTTG

86 BMEI1684 / DI019 **149** TGCTTGATTTCCTCCAATTGCTCATC GCTGCAGCTGAAAAGCTACGTATTGC

87 BMEI1685 **163** TTGATGGAATTGGTGGAGAGTTTTCG CAACCAGTCCGGTCTCCTTATGTTGG

88 BMEI1686 / DI021 **265** CGAAGGCGAAAAACTGGATA GGACCGCATGTTTACCTTTG

89 BMEI1687 / DI022 **167** CAGAAATCGAGCGCCTTATC TTAGCCCTTTGTCTGGATCG

90 BMEI1688 **431** ATGATTGCCTTGCAGACATTGC CTTTGCCAAGAGTACGGCTCAG

91 BMEI1689 / DI025 **271** GCGGTGTTGAAAATGGATGAAC ACCAAATCGGCGCATTCTATTC

92 BMEI1690 / DI026 **160** ACGCAGGAGGAAAGAACACC ACGCCATCATCTTCCTCAAG

93 BMEI1691 **206** GCATGCTTGCGACTGAAAC TAGTTCATCAGGACGCAACG

94 BMEI1692 / DI038 **201** AGTGATGCTCTCTTGCGACA ATCGGATGCAGCCTCAAG

95 BMEI1693 **151** GTTGAAGGCGCATCAAGAACAG GACGGTCAATGCGTGCTGATAC

96 BMEI1694 / DI042 **150** GGGACAAACTGAACGACGAT AATGAAAACGGGCTTCCAG

97 BMEI1695 **239** CTTTGTCGAAGGTGGTCACTGC AGCTTCATTCAACATCGCCGTA

98 BMEI1696 / DI052 **526** CCTTGCCGATCTGAACAATAGC ATCTGGAGAAAGCCTGAACACG

99 BMEI1697 / DI056 **857** GACAGCGGCCAGAGAGATTACC TGAGGATATCGTTCCAGTCTCG

100 BMEI1698 / DI057 **245** CTTTGTCGAAGGTGGTCACTGC TGATGGAGCTTCATTCAACATCG

101 BMEI1699 / DI058 **183** TCATGCTGTGCCTCCAATTCC TTGCTGAGCAGCAGCAAGAAC

102 BMEI1700 / DI059 **207** CTTTACTGGAGAGGGTGAACCAG CAAGTCACCGATCCTCTCCTG

103 BMEI1701 / DI060 **221** ATGTGCCTGACAAGCTCAGATG GATGAAGCCTTTGGCCTTAAAC

104 BMEI1702 / DI061 **169** TTATGCGACCTTGACTGACG GTCATTCGTTGCATCCTTCC
